# Rituximab versus azathioprine for maintenance of remission for patients with ANCA-associated vasculitis and relapsing disease: an international randomised controlled trial

**DOI:** 10.1136/ard-2022-223559

**Published:** 2023-03-23

**Authors:** Rona M Smith, Rachel B Jones, Ulrich Specks, Simon Bond, Marianna Nodale, Reem Al-jayyousi, Jacqueline Andrews, Annette Bruchfeld, Brian Camilleri, Simon Carette, Chee Kay Cheung, Vimal Derebail, Tim Doulton, Alastair Ferraro, Lindsy Forbess, Shouichi Fujimoto, Shunsuke Furuta, Ora Gewurz-Singer, Lorraine Harper, Toshiko Ito-Ihara, Nader Khalidi, Rainer Klocke, Curry Koening, Yoshinori Komagata, Carol Langford, Peter Lanyon, Raashid Luqmani, Carol McAlear, Larry W Moreland, Kim Mynard, Patrick Nachman, Christian Pagnoux, Chen Au Peh, Charles Pusey, Dwarakanathan Ranganathan, Rennie L Rhee, Robert Spiera, Antoine G Sreih, Vladamir Tesar, Giles Walters, Caroline Wroe, David Jayne, Peter A Merkel

**Affiliations:** 1 Medicine, University of Cambridge, Cambridge, UK; 2 Renal Medicine, Addenbrooke's Hospital, Cambridge, UK; 3 Pulmonary Medicine, Mayo Clinic, Rochester, Minnesota, USA; 4 Cambridge Clinical Trials Unit, Cambridge University Hospitals NHS Foundation Trust, Cambridge, UK; 5 Nephrology, University Hospitals of Leicester NHS Trust, Leicester, UK; 6 NIHR Leeds Musculoskeletal Biomedical Research Unit, Leeds Teaching Hospitals Trust, Leeds, UK; 7 Nephrology, Karolinska University Hospital and Karolinska Institute, Stockholm, Sweden; 8 Nephrology, Ipswich Hospital NHS Trust, Ipswich, UK; 9 Rheumatology, University of Toronto, Toronto, Ontario, Canada; 10 Nephrology, University of Leicester, Leicester, UK; 11 Medicine, University of North Carolina at Chapel Hill, Chapel Hill, North Carolina, USA; 12 Nephrology, East Kent Hospitals University NHS Foundation Trust, Canterbury, UK; 13 Nephrology, Nottingham University Hospitals NHS Trust, Nottingham, UK; 14 Rheumatology, Cedars-Sinai Medical Center, Los Angeles, California, USA; 15 Hemovascular Medicine and Artificial Organs, Faculty of Medicine, University of Miyazaki, Miyazaki, Japan; 16 Allergy and Clinical Immunology, Chiba University, Chiba, Japan; 17 Rheumatology, University of Michigan, Ann Arbor, Michigan, USA; 18 Nephrology, University of Birmingham, Birmingham, UK; 19 The Clinical and Translational Research Center, Kyoto Prefectural University of Medicine, Kyoto, Japan; 20 Medicine, McMaster University, Hamilton, Ontario, Canada; 21 Rheumatology, Dudley Group of Hospitals NHS Trust, Dudley, UK; 22 Rheumatology, The University of Utah, Salt Lake City, Utah, USA; 23 First Department of Internal Medicine, Kyorin University School of Medicine, Tokyo, Japan; 24 Rheumatic and Immunologic Diseases, Cleveland Clinic Foundation, Cleveland, Ohio, USA; 25 Rheumatology, Nottingham University Hospital, Nottingham, UK; 26 Nuffield Department of Orthopaedics, Rheumatology and Musculoskeletal Science (NDORMs), University of Oxford, Oxford, UK; 27 Rheumatology, University of Pennsylvania Perelman School of Medicine, Philadelphia, Pennsylvania, USA; 28 Medicine/Rheumatology, University of Pittsburg, Pittsburg, Pennsylvania, USA; 29 Vasculitis and lupus clinic, Cambridge University Hospitals NHS Foundation Trust, Cambridge, UK; 30 UNC Kidney Center, Division of Nephrology and Hypertension, University of North Carolina at Chapel Hill, Chapel Hill, North Carolina, USA; 31 Mount Sinai Hospital, University of Toronto, Toronto, Ontario, Canada; 32 Nephrology, Royal Adelaide Hospital, Adelaide, South Australia, Australia; 33 Medicine, Imperial College London, London, UK; 34 Medicine, Royal Brisbane and Women's Hospital, Herston, Queensland, Australia; 35 Rheumatology, Perelman School of Medicine at the University of Pennsylvania, Philadelphia, Pennsylvania, USA; 36 Rheumatology, Hospital for Special Surgery, New York, New York, USA; 37 Medicine, Charles University, Praha, Czech Republic; 38 Nephrology, Australian National University Medical School, Canberra, Australian Capital Territory, Australia; 39 Nephrology, South Tees Hospitals NHS Foundation Trust, Middlesbrough, UK; 40 Medicine, Addenbrooke's Hospital, Cambridge, UK; 41 Medicine, University of Pennsylvania, Philadelphia, Pennsylvania, USA

**Keywords:** rituximab, therapeutics, systemic vasculitis, B-lymphocytes, granulomatosis with polyangiitis

## Abstract

**Objective:**

Following induction of remission with rituximab in anti-neutrophil cytoplasmic antibody-associated vasculitis (AAV) relapse rates are high, especially in patients with history of relapse. Relapses are associated with increased exposure to immunosuppressive medications, the accrual of damage and increased morbidity and mortality. The RITAZAREM trial compared the efficacy of repeat-dose rituximab to daily oral azathioprine for prevention of relapse in patients with relapsing AAV in whom remission was reinduced with rituximab.

**Methods:**

RITAZAREM was an international randomised controlled, open-label, superiority trial that recruited 188 patients at the time of an AAV relapse from 29 centres in seven countries between April 2013 and November 2016. All patients received rituximab and glucocorticoids to reinduce remission. Patients achieving remission by 4 months were randomised to receive rituximab intravenously (1000 mg every 4 months, through month 20) (85 patients) or azathioprine (2 mg/kg/day, tapered after month 24) (85 patients) and followed for a minimum of 36 months. The primary outcome was time to disease relapse (either major or minor relapse).

**Results:**

Rituximab was superior to azathioprine in preventing relapse: HR 0.41; 95% CI 0.27 to 0.61, p<0.001. 19/85 (22%) patients in the rituximab group and 31/85 (36%) in the azathioprine group experienced at least one serious adverse event during the treatment period. There were no differences in rates of hypogammaglobulinaemia or infection between groups.

**Conclusions:**

Following induction of remission with rituximab, fixed-interval, repeat-dose rituximab was superior to azathioprine for preventing disease relapse in patients with AAV with a prior history of relapse.

**Trial registration number:**

NCT01697267; ClinicalTrials.gov identifier

**WHAT IS ALREADY KNOWN ON THIS TOPIC?**

- Rituximab is superior to azathioprine for the prevention of major relapse following cyclophosphamide induction therapy in anti-neutrophil cytoplasmic antibody-associated vasculitis (AAV).

**WHAT THIS STUDY ADDS?**

- These data confirm the place of rituximab as the standard of care for maintenance therapy. But, despite a higher dose rituximab regimen, relapses still occurred during treatment, and there was an increased risk of relapse after stopping rituximab.

**HOW THIS STUDY MIGHT AFFECT RESEARCH, PRACTICE OR POLICY?**

- The ongoing relapse risk together with associated safety concerns of extended rituximab therapy illustrate the need for newer therapeutic agents in AAV.

## Background

Granulomatosis with polyangiitis (GPA) and microscopic polyangiitis (MPA) are the major subgroups of anti-neutrophil cytoplasmic antibody (ANCA)-associated vasculitis (AAV).^1^ Untreated, AAV has a mortality of 93% within 2 years, primarily due to renal and respiratory failure.^2^ The introduction of glucocorticoids and cyclophosphamide improved survival, inducing remission at 1 year in 80% of patients. B-lymphocytes contribute to the pathogenesis of AAV and rituximab is an effective therapy for induction of remission and is superior to cyclophosphamide for the treatment of relapsing disease.^3 4^ However, over 50% of patients relapse within 5 years of diagnosis,^5–7^ including after induction of remission with rituximab, especially in patients with a history of relapse.^8–10^ Relapses reflect further episodes of inflammation and contribute to irreversible tissue damage, end-stage kidney failure, treatment-related toxicity, chronic morbidity, increased mortality and high health-related costs. More-effective strategies to prevent relapse in AAV are needed.

Fixed-interval, repeat dose rituximab was superior to azathioprine as a maintenance strategy in a largely newly diagnosed AAV after induction with cyclophosphamide and glucocorticoid in the MAINRITSAN 1 trial.^11^ However, prolonged use of rituximab in AAV has been associated with an increased risk of infection and the development of hypogammaglobulinaemia.^12^ The optimal strategy to maintain remission following induction of remission with rituximab, especially for treatment of relapse, remains unclear.

RITAZAREM was an international, randomised, controlled trial designed to assess whether fixed-interval rituximab was superior to azathioprine for the maintenance of remission following induction of remission with rituximab and glucocorticoids in patients with relapsing AAV. Furthermore, it was hypothesised that increased doses of rituximab would reduce the risk of relapse beyond the maintenance treatment period.

## Methods

### Study design

The RITAZAREM trial had three phases. The protocol design and results of the induction phase have been reported.^13 14^

1. *Induction phase* (enrolment through month 4): induction therapy comprised of rituximab (four doses of 375 mg/m^2^/week) and oral prednisone/prednisolone commencing at either 1.0 mg/kg/day (high dose) or 0.5 mg/kg/day (low dose), both reducing to 10 mg/day or less, selected at physician discretion. Intravenous methylprednisolone up to a cumulative dose of 3000 mg was permitted in the 2 weeks before or 1 week after enrolment.^13 14^
2. *Maintenance phase*: (4–24 months from enrolment). Patients who had achieved remission, defined as a Birmingham Vasculitis Activity Score for Wegener’s granulomatosis (BVAS/WG) ≤1 and prednisone/prednisolone dose ≤10 mg/day, were randomised to receive rituximab or azathioprine.
3. *Follow-up phase:* this off-treatment phase commenced after completion of the maintenance phase at month 24 and lasted for a further 12–24 months (36–48 months from enrolment).

This paper reports the results of the maintenance and follow-up phases of the trial.

### Patients

Patients were aged over 15 years and had a diagnosis of GPA or MPA according to the Chapel Hill Consensus Conference 2012 definitions, and a current or prior positive test for proteinase 3 (PR3)-ANCA or myeloperoxidase (MPO)-ANCA.^15^ All patients had disease relapse defined by one major or three minor item of disease activity on the BVAS/WG after achieving remission following therapy with a combination of glucocorticoids and an immunosuppressive agent. Patients with other multisystem autoimmune diseases were excluded.

Patients were recruited from 29 centres in seven countries between April 2013 and November 2016. The last patient visit was in November 2019.

### Randomisation and masking

RITAZAREM was an open-label, unblinded study. Patients who achieved disease remission by month 4 were randomised into the maintenance phase using a web-based system in a 1:1 ratio, to receive rituximab or azathioprine. They were stratified at randomisation according to:

1. ANCA type: PR3-ANCA or MPO-ANCA.
2. *Relapse type: severe or non-severe*. A severe relapse was defined as the development of a new or recurrent item of major disease activity on BVAS/WG.^16^ A non-severe relapse was any increase in disease activity that did not meet the definition of a severe relapse.
3. *Oral glucocorticoid induction regimen: high or low dose.*

### Maintenance phase interventions

#### Rituximab

Intravenous rituximab 1000 mg repeated every 4 months for five doses (months 4, 8, 12, 16 and 20 from enrolment). This dose was based on prior observational studies and the interval was designed to minimise the risk of relapse.^10^ Rituximab was withheld for plasma IgG <3 g/L and could be recommenced at the next treatment time point if plasma IgG >3 g/L.

#### Azathioprine

Oral azathioprine 2 mg/kg/day for 24 months, then reduced by 50% and withdrawn at month 27. Patients intolerant to azathioprine received either methotrexate (oral or subcutaneous), 25 mg/week, if their estimated glomerular filtration rate (eGFR) was >50 mL/min, or mycophenolate mofetil 2 g/day, if their eGFR was ≤50 mL/min.

#### Glucocorticoids

A prednisone/prednisolone dose of 10 mg/day or less was a requirement for randomisation at month 4. This dose was reduced to 5 mg/day by month 6 through to month 16 then reduced to 2.5 mg/day and withdrawn at month 20 (online supplemental eTable 1). Patients experiencing a first minor relapse received an increase in oral prednisone/prednisolone to 20 mg/day reducing over 6 weeks to their dose prior to the relapse and continued their other immunosuppressive agent (rituximab or azathioprine). After a second minor or first major relapse treatment was according to physician discretion.

10.1136/ard-2022-223559.supp5 Supplementary data

#### Other treatments

Medications to prevent *pneumocystis (carinii) jiroveci* infection and/or to prevent osteoporosis were prescribed according to local practice.

#### Assessments

Evaluations, including clinical, laboratory and patient-reported outcomes, were performed at months 4, 8, 12, 16, 20, 24, 27, 30, 36, 42 and 48. The common closeout date for those patients remaining in the trial was when the final patient reached month 36.

#### Outcomes

The primary outcome was time from randomisation to disease relapse, defined as the return or first appearance of at least one item on BVAS/WG. Major relapse required at least one major BVAS/WG item. Relapses were reviewed by a blinded adjudication committee. Secondary outcomes included the proportions who maintained remission at the end of the maintenance phase, or end of the follow-up phase; time to major relapse; cumulative accrual of damage measured by the Combined Disease Assessment instrument^17^ (online supplemental eTable 2); cumulative glucocorticoid exposure; health-related quality of life measures using the SF-36; rates of serious adverse events (SAEs), hypogammaglobulinaemia, defined as plasma IgG<5 g/L and infections.

#### Compliance

Compliance for the rituximab group was defined as receipt of five doses of rituximab (unless withheld for IgG <3 g/L) and no oral immunosuppressive agents administered. Compliance in the azathioprine group was defined as ongoing receipt of azathioprine, methotrexate or mycophenolate mofetil between months 4 and 24.

### Statistical analyses

#### Sample size calculation

Enrolment continued until at least 160 patients were randomised. This sample size was calculated based on a goal of achieving 90% power under the alternative hypothesis of a HR of 0.42 at the 5% significance level with 58 observed relapses. This assumed a drop-out rate of 5% at 24 months and a relapse-free rate of 75% and 50% at 48 months in the rituximab and azathioprine arms, respectively.

### Analysis

Results are reported for the 170 randomised patients, except for safety parameters for which data on all 188 enrolled patients are presented. The primary intention-to-treat analysis was based on a Cox proportional hazard model, adjusted for the stratification factors (ANCA type, relapse severity and prednisone induction regimen) for the difference in the distribution of relapse-free survival between the rituximab and azathioprine groups with a closed testing procedure. First, the null hypothesis was tested for a HR of 1 at all time points. If this was rejected at a 5% level, then two further subhypotheses were prespecified using time-varying covariates; up to 24 months and after 24 months. Hazard ratios and 95% CIs were reported. A p value less than 5% was considered statistically significant.

Kaplan-Meier estimates for relapse-free survival at 24 and 48 months and median relapse-free survival with the corresponding 95% CIs by treatment allocation are also presented. Multivariable analysis was performed on risk factors for the development of hypogammaglobulinaemia. Data were analysed using R V.3.6.1.

## Results

### Patient demographics

**Figure 1:**
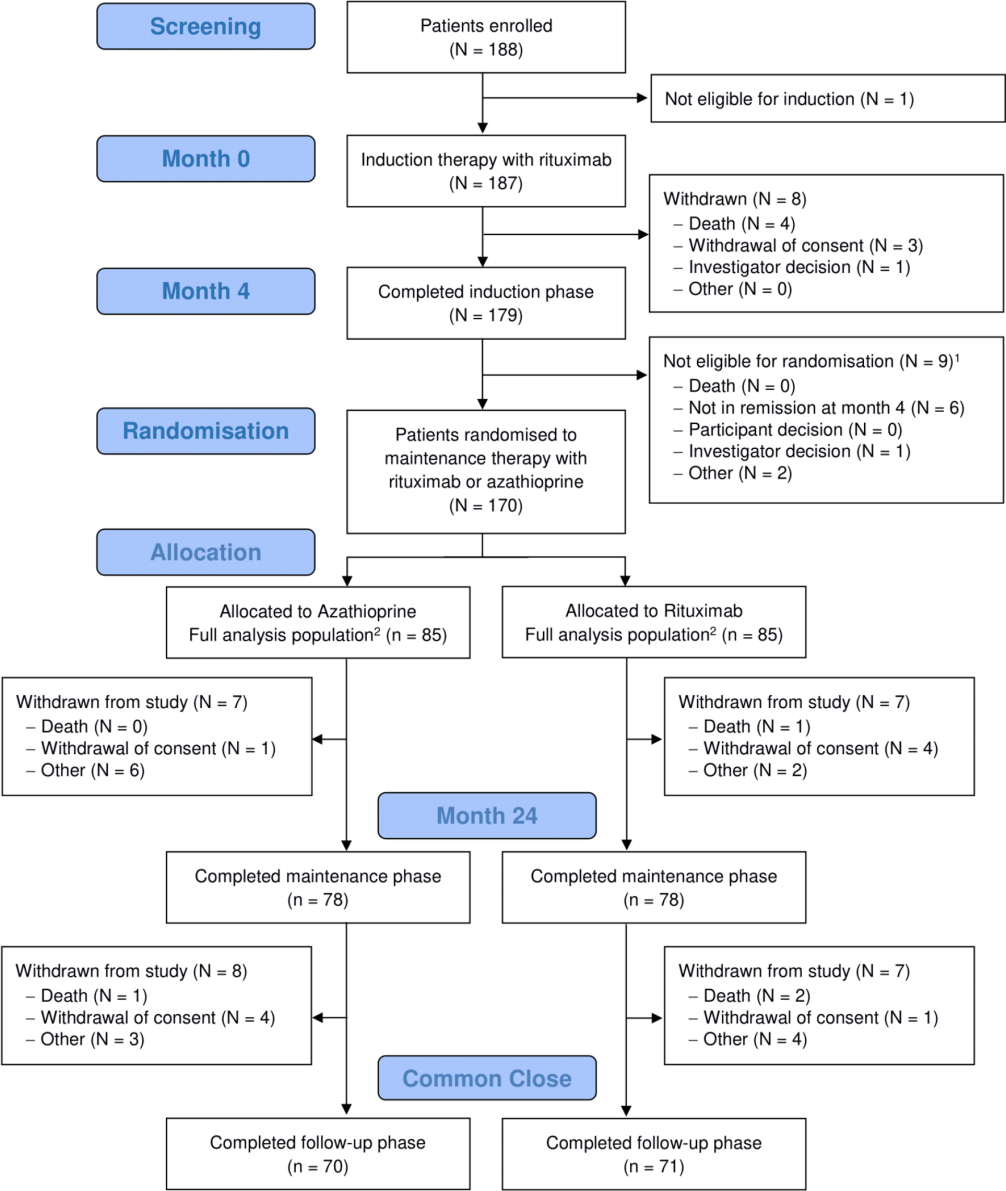
Consort Diagram for the RITAZAREM trial. ^1^Patients not eligible for randomisation remain under long-term follow-up, unless they withdraw consent. ^2^The full analysis population includes all randomised patients, including those subsequently withdrawn.

One hundred and eighty-eight patients were enrolled and received induction therapy with rituximab and glucocorticoids.^16^ 170 (90%) were randomised at month 4 to rituximab (N=85) or azathioprine (N=85) treatment groups and were the study population for the current analysis (figure 1 and table 1). One-hundred and twenty-three (72%) had PR3-ANCA, and 47 (38%) had MPO-ANCA. One-hundred and six (62%) patients had at least one major disease activity item, and 48 (28%) received the high-dose glucocorticoid induction regimen. The disposition of the enrolled patients is detailed in figure 1.

**Table 1:** Baseline demographics of randomised study population in the RITAZAREM trial

	Total (N=170)	Rituximab (N=85)	Azathioprine (N=85)
Age, years: mean (SD)	57.8 (14.5)	57.1 (15.1)	58.6 (13.9)
Male, number (%)	84 (49%)	43 (51%)	41 (48%)
Race, number (%)			
White	155 (91%)	78 (92%)	77 (91%)
Asian	10 (6%)	5 (6%)	5 (6%)
Hispanic	3 (2%)	2 (2%)	1 (1%)
Black	0	0	0
Other	2 (1%)	0	2 (2%)
Disease duration, years: mean (SD)	7.16 (6.52)	7.38 (6.94)	6.93 (6.10)
Prior treatment with cyclophosphamide			
Number of patients (%)	133 (78%)	67 (79%)	66 (78%)
Cumulative dose, grams (g): mean (SD)	25.7 (43.3)	24.4 (50.4)	26.9 (35.5)
Prior rituximab therapy			
Number of patients (%)	60 (35%)	33 (39%)	27 (32%)
Cumulative dose, grams (g): mean (SD)	4.88 (3.24)	4.47 (2.95)	5.40 (3.57)
Glucocorticoid induction regimen			
1 mg/kg/day starting dose (1A)	48 (28%)	24 (28%)	24 (28%)
0.5 mg/kg/day starting dose (1B)	122 (72%)	61 (72%)	61 (72%)
ANCA type			
Anti-proteinase 3	123 (72%)	61 (72%)	62 (73%)
Anti-myeloperoxidase	47 (28%)	24 (28%)	23 (27%)
Relapse type on entry into trial			
Severe	106 (62%)	52 (61%)	54 (64%)
Non-severe	64 (38%)	33 (39%)	31 (36%)

### Efficacy

**Figure 2:**
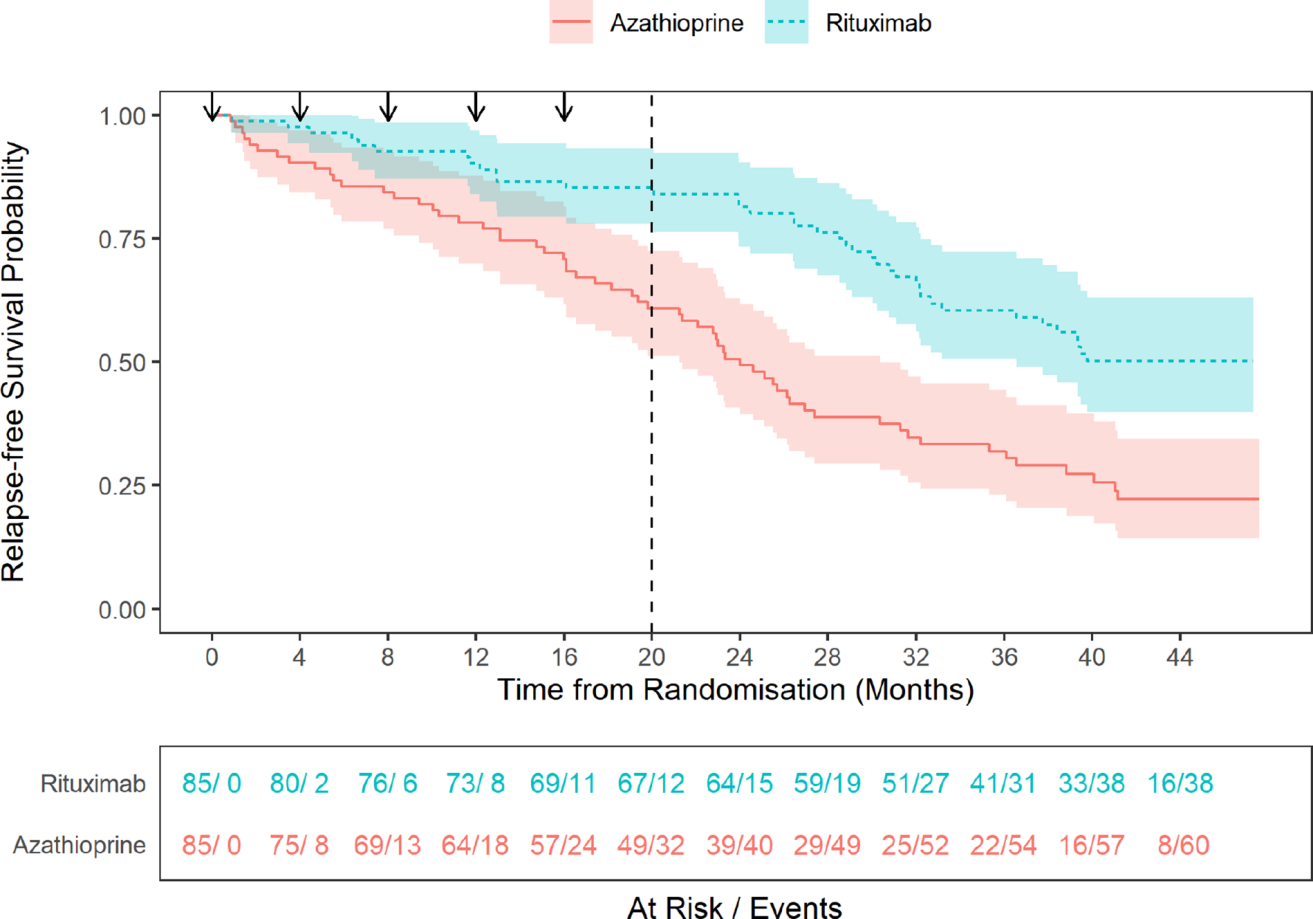
Probability of relapse-free survival: rituximab compared with azathioprine. Black arrows represent 1000 mg dose of rituximab. Dashed vertical line indicates end of maintenance treatment period and start of the follow-up period per protocol. Shaded areas represent 95% CIs.

Over the combined maintenance and follow-up phases, rituximab was superior to azathioprine for the prevention of major or minor disease relapse: HR 0.41, 95% CI 0.27 to 0.61, p<0.001 (figure 2). The HR during the maintenance phase was 0.35, 95% CI 0.18 to 0.66, p=0.001, and during the follow-up phase was 0.45, 95% CI 0.26 to 0.78, p=0.004.

Thirty-eight of 85 (45%) patients in the rituximab group experienced 52 relapses, 11 major and 41 minor. Sixty of 85 (71%) patients in the azathioprine group experienced 89 relapses, 28 major and 61 minor. The overall HR for major relapse was 0.36, 95% CI 0.18 to 0.73, p=0.004. During the maintenance phase, 13/85 (15%) in the rituximab group relapsed compared with 32/85 (38%) in the azathioprine group. At month 24, the relapse-free survival rate was 0.85, 95% CI 0.78 to 0.93, for the rituximab compared with 0.61, 95% CI 0.51 to 0.73, for the azathioprine groups. During the follow-up phase, there were 33 relapses in 25 from the rituximab compared with 49 relapses in 28 in the azathioprine groups. Five patients in the rituximab group experienced a major relapse during the follow-up period compared with 11 in the azathioprine group. At month 48, the rate for continued remission was 0.50, 95% CI 0.40 to 0.63, for the rituximab and 0.22, 95% CI 0.14 to 0.35, for the azathioprine groups.

**Figure 3:**
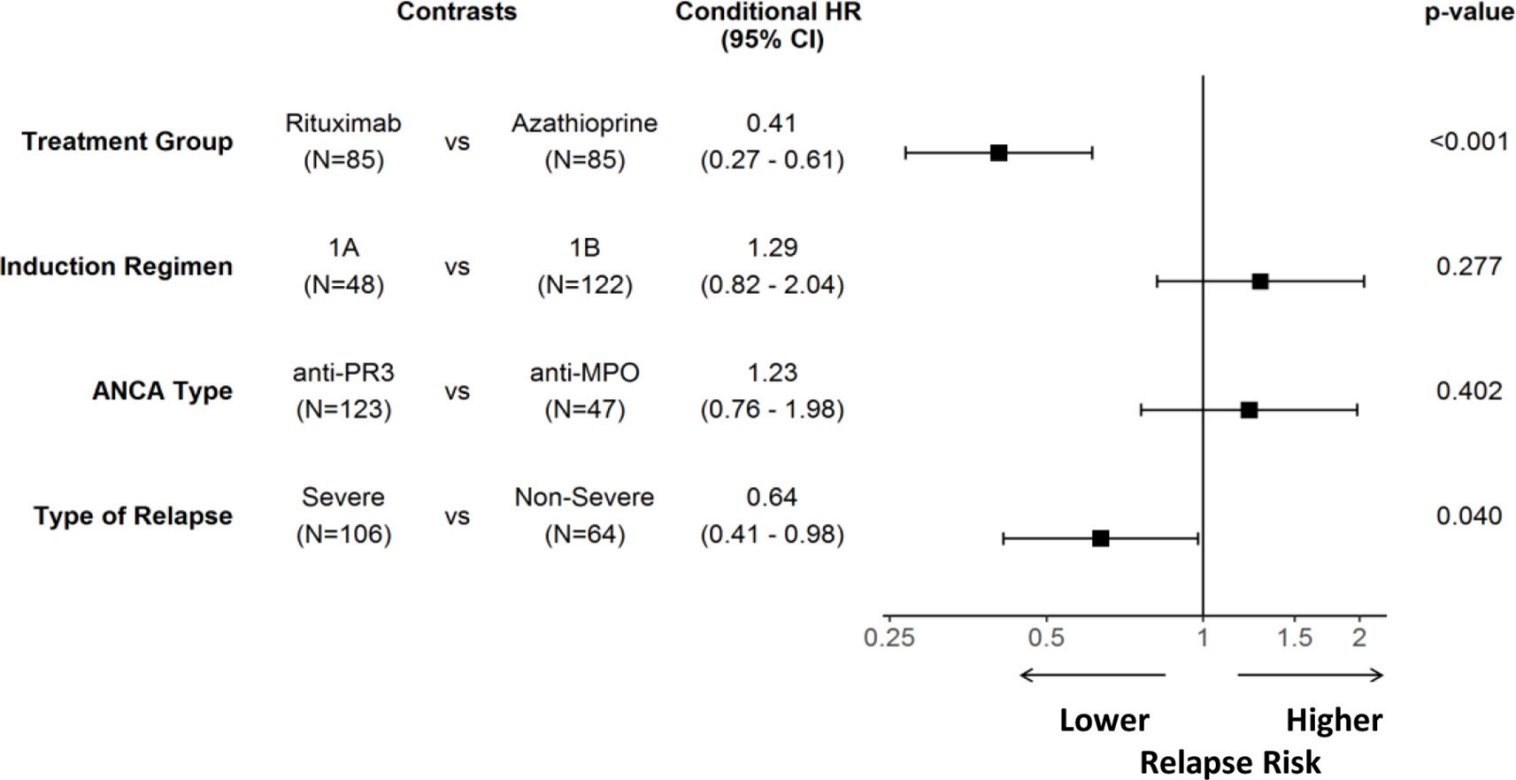
Multivariate model of clinical predictors for relapse in the RITAZAREM trial. Induction regimen refers to glucocorticoid dose. 1A—1 mg/kg/day starting dose (maximum 60 mg daily); 1B—0.5 mg/kg/day starting dose (maximum 30 mg daily). PR3, proteinase 3; MPO, myeloperoxidase. The estimates are from a multiple regression model that simultaneously adjusts for the treatment and all covariates.

In a multiple regression model, neither the glucocorticoid induction regimen (high or low dose) nor ANCA subtype (PR3-ANCA or MPO-ANCA) influenced relapse risk: HR 1.29, 95% CI 0.82 to 2.04, p=0.277 and HR 1.23, 95% CI 0.76 to 1.98, p=0.402, respectively (online supplemental eFigure 1). Individuals who entered the trial with major BVAS/WG items at relapse were less likely to experience a disease relapse during the trial: HR 0.64, 95% CI 0.41 to 0.98, p=0.040 (figure 3).

10.1136/ard-2022-223559.supp1 Supplementary data

### Compliance with treatment per protocol

81/85 (95%) patients in the rituximab group were compliant and 78/85 (92%) in the azathioprine group. During the follow-up phase, 10/85 (12%) patients in the rituximab and 15/85 (18%) patient in the azathioprine groups continued immunosuppression. When the 11 patients non-compliant during the treatment period with the protocol-defined immunosuppressive therapy were excluded in the analysis, the HR for relapse was 0.38, 95% CI 0.25 to 0.58, p<0.001. When the 25 patients non-complaint during either the treatment or the follow-up phase were excluded in the analysis, the HR for relapse was 0.36, 95% CI 0.23 to 0.57, p<0.001.

The median cumulative prednisolone dose during the maintenance phase was identical in both groups (median 2100 mg, ranges 0–5700 mg in rituximab and 0–9000 mg in the azathioprine groups), deviations from the protocol were common, 44 (52%) in the rituximab and 57 (67%) in the azathioprine groups reported at least one deviation during the trial. At month 24, by which time the protocol-required cessation of glucocorticoids, 22/77 (29%) in the rituximab and 35/76 (46%) in the azathioprine groups were still receiving glucocorticoids, mean daily doses=2.28 mg (SD=5.45) for the rituximab and 2.8 mg (SD 5.5) for the azathioprine groups.

### Damage assessment

There was no difference in the accrual of damage between groups. The modified Combined Damage Assessment score increased by a mean of 0.571 (SD 0.909) and 1.09 (SD 1.18) in the rituximab group compared with 0.533 (SD 0.777) and 1.38 (SD 1.65) in the azathioprine group during the maintenance phase and whole trial, respectively.

### Quality of life measures

No differences were observed between study groups in any domains of the SF-36 score. In the rituximab and azathioprine groups, median physical component scores at randomisation were reduced at 37.25 (range 2.8–61.6) and 36.5 (1.5–58.1) and median mental component scores were 54.55 (19.6–67.7) and 53.8 (16.7–72.6). Scores remained stable across the trial, both during the maintenance and follow-up phases (online supplemental eFigure 2).

### CD19-positive B cells

During the maintenance phase, the median CD19 cell counts were 0×10^9^ /L (0–3) in the rituximab group and 0×10^9^ /L (0–5) in the azathioprine group. The median percentage of CD19 cells remained zero (0–5) in the rituximab group but increased to 0.1% (0–29) at month 12 and 0.3% (0–35.1) at month 24 in the azathioprine group. During the follow-up phase, the median CD19 cell counts were lower in the azathioprine group, this was confounded by the use of rituximab to treat relapses in this group (online supplemental eFigure 3).

10.1136/ard-2022-223559.supp3 Supplementary data

### Safety

Sixty-nine SAEs occurred in 37 (44%) patients in the rituximab and 105 in 48 (56%) in the azathioprine groups (table 2). There was no difference in time to first SAE between groups (online supplemental eFigure 4). Nineteen (22%) patients in the rituximab and 31 (36%) patients in the azathioprine groups experienced at least one SAE during the treatment period. Nineteen severe infections occurred in 15 (18%) patients in the rituximab and 27 in 19 (22%) patients in the azathioprine groups (online supplemental eTable 3). One-hundred and ninety-seven and 207 non-severe infections occurred in 54 (64%) and 62 (73%) patients in the rituximab and azathioprine groups, respectively. One case of progressive multifocal leukoencephalopathy occurred after the induction period in a patient not randomised into the maintenance phase of the trial. Thirty-six (42%) patients in the rituximab group had a plasma IgG level <5 g/L at some point during the trial and 8 (9%) had a plasma IgG level <3 g/L compared with 26 (31%) and 6 (7%) patients in the azathioprine group. A lower plasma IgG level at baseline (OR 0.52 baseline IgG; 95% CI 0.40 to 0.65, p<0.001) and high-dose glucocorticoids during induction (OR 8.6; 95% CI 3.02 to 27.58, p<0.001) were associated with the development of hypogammaglobulinaemia (table 3). One patient, from the rituximab group, received intravenous immunoglobulin during the trial for treatment of hypogammaglobulinaemia and repeated infections. Eleven patients developed a new malignancy during the trial: five in the rituximab (skin (2), prostate (1), pancreas (1), oesophagus (1)) and six in the azathioprine groups (skin (5), pancreas (1)). Four patients died during the trial; three in the rituximab (infection (1), malignancy (1), other (1)) and one in the azathioprine groups (malignancy).

10.1136/ard-2022-223559.supp4 Supplementary data

**Table 2:** Adverse events according to treatment regimen in the RITAZAREM trial

	Total (N=188)	Rituximab (N=85)	Azathioprine (N=85)	Not randomised (N=18)
Number (%) of patients with a serious adverse event	92 (49%)	37 (44%)	48 (56%)	7 (39%)
Number (%) of patients with a serious infection	39 (21%)	15 (18%)	19 (22%)	5 (28%)
Number (%) of patients with a non-serious infection	119 (63%)	54 (64%)	62 (73%)	3 (17%)
Number (%) of patients with plasma IgG<5 g/L	66 (35%)	36 (42%)	26 (31%)	4 (22%)
Number (%) of patients with plasma IgG<3 g/L	17 (9%)	8 (9%)	6 (7%)	3 (17%)

**Table 3:** Multivariable model of predictors for the development of hypogammaglobulinaemia in the RITAZAREM trial

Variable	OR	95% CI	P value
Maintenance treatment (rituximab vs azathioprine)	2.2	(0.87 to 5.72)	0.104
Glucocorticoid induction regimen (1A vs 1B)	8.6	(3.02 to 27.58)	<0.001
ANCA status at enrolment (anti-PR3 vs anti-MPO)	0.78	(0.27 to 2.26)	0.639
Type of relapse (severe vs non-severe)	2.2	(0.82 to 6.07)	0.124
Previous rituximab (yes vs no)	1.2	(0.41 to 3.53)	0.740
Previous cyclophosphamide (yes vs no)	1.1	(0.20 to 6.36)	0.944
Previous rituximab or cyclophosphamide (yes vs no)	7.3	(0.57 to 128.37)	0.144
Baseline plasma IgG (g/L)*	0.52	(0.40 to 0.65)	<0.001
Intercept	0.013	(0.00 to 0.12)	–

Glucocorticoid induction regimen: 1A=1 mg/kg/day starting dose; 1B=0.5 mg/kg/day starting dose.

*Baseline plasma IgG level was centred around the mean value (9.56). The intercept gives an estimate of the absolute odds for a patient with the mean value of baseline IgG, and the reference levels of the other binary predictors.

MPO, myeloperoxidase; PR3, proteinase 3.

## Discussion

This international, randomised, controlled trial demonstrated that rituximab was superior to azathioprine for prevention of disease relapse in patients with AAV with a prior history of relapse, following reinduction of remission with rituximab and glucocorticoids, and there was lower average glucocorticoid exposure in the rituximab group.

The MAINRITSAN 1 trial demonstrated the superiority of rituximab over azathioprine for the prevention of relapse following induction of remission with cyclophosphamide in a study population with predominantly newly diagnosed AAV patients. The higher relapse rate found in this trial compared with the MAINRITSAN 1 trial reflects differences in patient populations and trial design.^11^ RITAZAREM recruited patients at relapse, which is associated with a higher subsequent relapse risk. Both major and minor relapses were reported as part of the primary endpoint of relapse-free survival in RITAZAREM, reflecting the importance of minor relapses in cumulative treatment exposure, and the follow-up period was longer, at 48 months. The cumulative rituximab dose during the maintenance phase, 5000 mg, was double that used in MAINRITSAN, yet relapses were still seen in 15% of the rituximab group during treatment, identifying a subset of patients with disease refractory to higher dose rituximab. The lower relapse risk in those with major BVAS/WG items at enrolment is consistent with previous observations of lower relapse risk with worse renal vasculitis.^18^ Furthermore, after discontinuation of therapy, relapses were frequent in both groups indicating that the benefit of rituximab, even at a high dose, was not sustained beyond the treatment period.

SAEs and infections were common, consistent with previous studies in AAV, and there were no new safety signals for these medications in this population. Hypogammaglobulinaemia, secondary immunodeficiency and impaired vaccine responses, is a concern with use of repeated doses of rituximab. Although median plasma IgG levels were stable in both the rituximab and azathioprine groups across the trial, 42% of patients in the rituximab group and 31% in the azathioprine group developed a plasma IgG level <5 g/L; however, it should be noted that all patients had received rituximab induction at trial entry. In RITAZAREM, higher glucocorticoid exposure and lower baseline plasma IgG levels were associated with the development of hypogammaglobulinaemia, a finding consistent with a prior reports.^12 19^ In the context of the COVID-19 pandemic, poorer vaccine responses, both in term of absolute antibody titres and neutralising capacity, when compared with non-B cell depleting immunosuppressive agents, have been observed in several cohorts despite booster vaccine doses and are important consideration when making therapeutic decisions.^20–26^

The strengths of this study include this being the largest cohort of patients with relapsing AAV recruited into a clinical trial, centralised randomisation and recruitment from 29 centres across four continents, minimising centre or regional bias. The study had low rates of treatment crossover and a long period of follow-up after trial medications were discontinued, a design aimed at detecting the prolonged effects or safety issues of the interventions.

The study was limited by use of open-label trial medication, but potential impact on trial end-points was counterbalanced by a blinded adjudication end-point committee. Extended use of glucocorticoids was employed due to their known impact on relapse risk and the inclusion of a population at high risk of relapse, but their value could not be assessed and there remains a need to minimise glucocorticoids among patients with relapsing disease who have already accrued considerable exposure to glucocorticoids.^27^ Prior immunosuppressive exposure may have potentially confounded the results, but exposures were comparable across the treatment groups.

In conclusion, the results of the RITAZAREM trial show that repeat-dose rituximab is more effective than azathioprine for prevention of relapse for patients with AAV with relapsing disease induced with rituximab and glucocorticoids. These data extend previous reports on the efficacy of rituximab for induction of remission for relapsing disease and confirms the place of rituximab as the standard of care for maintenance therapy. The results should also prompt further reductions in glucocorticoid exposure for AAV.^28^ Despite a higher dose rituximab regimen than previously studied, relapses still occurred, and this, together with the increased risk of relapse after stopping rituximab, and the associated safety risks, illustrate the need for newer therapeutic agents for AAV. No new safety signals were seen with rituximab, and infections and hypogammaglobulinaemia remaining common problems in this patient population. Future treatment strategies for AAV may necessitate a more individualised approach, taking into account the risk of relapse balanced against the risk of adverse events with extended treatment.^29^

10.1136/ard-2022-223559.supp2 Supplementary data

## Data Availability

Data are available upon reasonable request. Data are available on reasonable request. De-identified participant data can be requested from the corresponding author.
