# Supplementary material for: Rituximab versus azathioprine for maintenance of remission for patients with ANCA-associated vasculitis and relapsing disease: an international randomised controlled trial
Source: Ann Rheum Dis. 2023 Mar 23;82(7):937–44. doi: 10.1136/ard-2022-223559 (PMC10313987; doi:10.1136/ard-2022-223559)
Supplement: Supplementary data [file ard-2022-223559supp005.pdf]

Online Material

Title: Rituximab versus azathioprine for maintenance of remission for patients with ANCA-associated vasculitis and relapsing disease: An international randomized controlled trial

eTable 1: Glucocorticoid dosing regimens used in the RITAZAREM trial

eTable 2: Calculation of modified Combined Damage Assessment Score

eTable 3: Line listing of Severe Infections in the RITAZAREM trial

eTable 1: Glucocorticoid dosing regimens used in the RITAZAREM trial

	Induction Schedule A (Prednisone/prednisolone 1 mg/kg group)		Induction Schedule B Prednisone/prednisolone 0.5 mg/kg group	
Time	<60 kg	≥60 kg	<60 kg	≥60 kg
Week 0	50	60	25	30
Week 2	35	45	20	25
Week 4	25	35	17.5	20
Week 6	20	25	15	17.5
Week 8	15	17.5	12.5	15
Week 10	12.5		12.5	
Week 12	10		10	
	Maintenance Schedule: Daily prednisone/prednisolone dose (mg)			
Week 16 (randomization)	10			
Week 18	7.5			
Week 22	5			
Month 16	2.5			
Month 20	0			

eTable 2: Calculation of modified Combined Damage Assessment Score

Category	Items	Maximum score
Musculoskeletal	1 - Significant muscle atrophy or weakness<br>2 - Deforming/erosive arthritis<br>3 - Osteoporosis/vertebral collapse<br>4 - Avascular necrosis<br>5 - Osteomyelitis	5
Skin / Mucous membranes	1 - Alopecia<br>2 - Cutaneous ulcers<br>3 - Mouth ulcers	3
Ocular	1 - Cataract<br>2 - Retinal changes or Retinal artery occlusion or Retinal vein occlusion<br>3 - Optical nerve atrophy<br>4 - Low vision or Diplopia<br>5 - Blindness in one eye (left or right)<br>6 - Blindness in second eye (both)<br>7 - Orbital wall destruction	7
Ear nose and throat (ENT)	1 - Sensorineural hearing loss or Conductive hearing loss<br>2 - Chronic rhinitis/crusting<br>3 - Nasal bridge collapse/saddle nose or Nasal septal perforation<br>4 - Chronic sinusitis<br>5 - Subglottic stenosis (no surgery)<br>6 - Subglottic stenosis (surgery)	6
Pulmonary	1 - Pulmonary hypertension<br>2 - Pulmonary fibrosis<br>3 - Pulmonary infarction<br>4 - Pleural fibrosis<br>5 - Chronic asthma<br>6 - Chronic breathlessness<br>7 - Irreversible loss of lung function	7
Cardiac	1 - Angina or Percutaneous coronary intervention<br>2 - Myocardial infarction<br>3 - Coronary artery bypass graft<br>4 - Cardiomyopathy (NYHA Class I/II or NYHA class III/IV)<br>5 - Valvular Disease<br>6 - Pericarditis or Pericardectomy<br>7 – Hypertension	7
Vascular	1 - Absent pulses in 1 limb<br>2 - 2nd episode of absent pulses in 1 limb<br>3 - Major vessel stenosis<br>4 - Claudication > 3 months<br>5 - Minor tissue loss<br>6 - Major tissue loss<br>7 - Subsequent major tissue loss<br>8 - Deep venous thrombosis or Complicated venous thrombosis	8
Gastrointestinal	1 - Gut infarction/resection<br>2 - Mesenteric insufficiency/pancreatitis<br>3 - Esophageal stricture/surgery<br>4 - Chronic peritonitis	4

Category	Items	Maximum score
Renal	1 - Estimated/measured GFR<50%<br>2 - Proteinuria (any item)<br>3 - End-stage renal disease or Dialysis	3
Neuropsychiatric	1 - Cognitive impairment<br>2 - Major psychosis<br>3 - Seizures<br>4 - Cerebrovascular accident<br>5 - 2nd cerebrovascular accident<br>6 - Cranial nerve lesion<br>7 - Sensory polyneuropathy or Motor Neuropathy<br>8 - Transverse myelitis	8
Other	1 - Gonadal failure (Premature ovarian failure or Azoospermia)<br>2 - Marrow failure<br>(Refractory cytopenia or Myelodysplastic syndrome)<br>3 - Diabetes mellitus<br>4 - Chemical cystitis<br>5 - Malignancy<br>6 - Other	6
TOTAL		64

eTable 3: Line listing of Severe Infections in the RITAZAREM trial

Infection type	Subjects Total	Azathioprine	Rituximab	Non-Randomized	Events Total
TOTAL	39	19	15	5	54
Appendicitis	3	1	2	0	3
Bronchitis	3	2	1	0	3
Cellulitis	1	1	0	0	1
Dacryocystitis	1	1	0	0	1
Diverticulitis	1	1	0	0	2
Gastroenteritis viral	1	1	0	0	1
Influenza	5	3	2	0	5
Lower respiratory tract infection	2	2	0	0	2
Peritonitis	1	1	0	0	2
Pneumonia	10	3	5	2	11
Pneumonia klebsiella	1	1	0	0	1
Pneumonia streptococcal	1	0	0	1	1
Pneumonia viral	1	0	1	0	1
Progressive multifocal leukoencephalopathy	1	0	0	1	1
Sepsis	2	0	2	0	2
Sinusitis	1	0	1	0	1
Urinary tract infection	3	2	1	0	3
Escherichia urinary tract infection	2	0	1	1	2
Periorbital abscess	1	0	1	0	1
Infective exacerbation of chronic obstructive airways disease	2	1	0	1	2
Pseudomonas infection	1	0	0	1	1
Respiratory tract infection	4	3	1	0	4
Metapneumovirus infection	1	0	1	0	1
Oral herpes	1	1	0	0	1
Systemic viral infection	1	0	0	1	1
