# Supplementary material for: Rituximab versus azathioprine for maintenance of remission for patients with ANCA-associated vasculitis and relapsing disease: an international randomised controlled trial
Source: Ann Rheum Dis. 2023 Mar 23;82(7):937–44. doi: 10.1136/ard-2022-223559 (PMC10313987; doi:10.1136/ard-2022-223559)
Supplement: Supplementary data [file ard-2022-223559supp001.pdf]

Supplementary Figure 1: Probability of relapse-free survival: rituximab compared to azathioprine according to ANCA sub-type: anti-MPO or anti-PR3

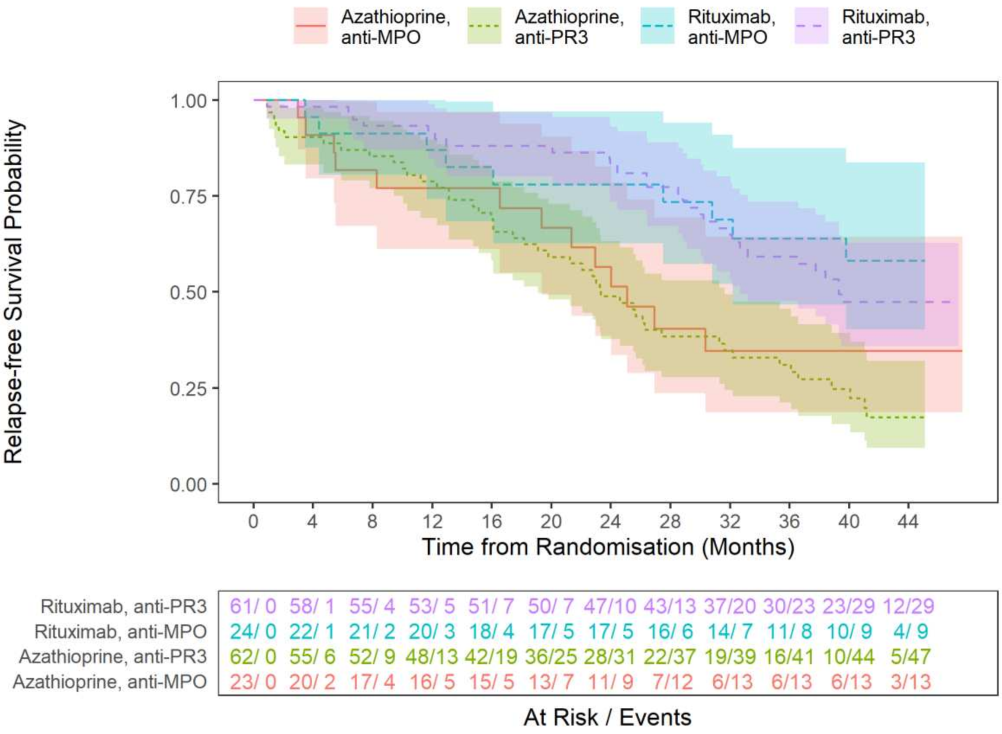

Shaded areas represent 95% confidence intervals.
