# Supplementary material for: Rituximab versus azathioprine for maintenance of remission for patients with ANCA-associated vasculitis and relapsing disease: an international randomised controlled trial
Source: Ann Rheum Dis. 2023 Mar 23;82(7):937–44. doi: 10.1136/ard-2022-223559 (PMC10313987; doi:10.1136/ard-2022-223559)
Supplement: Supplementary data [file ard-2022-223559supp003.pdf]

Supplementary Figure 3: Percentage of blood CD19 B cells throughout the RITAZAREM trial among patients in the two treatment groups

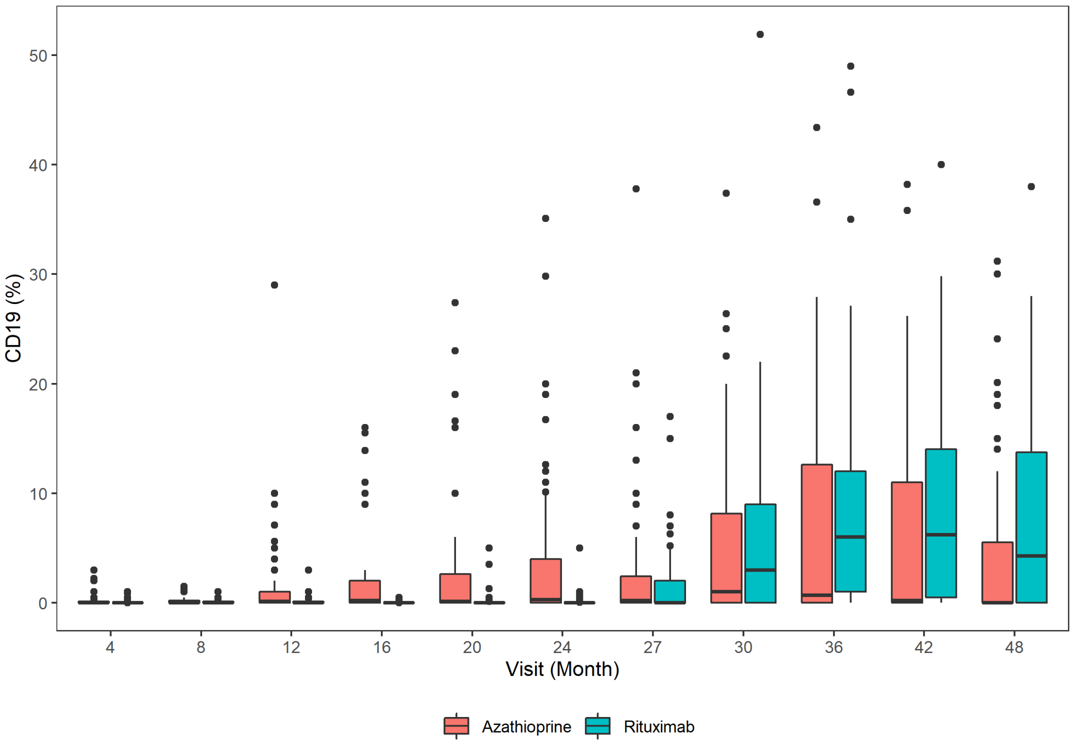

Boxes show the interquartile range (IQR) and horizontal line inside the box shows the median. The two whiskers on either side of the box represent values, up to and including, 1.5 times the length of the IQR. Values outside the range of the whiskers (more than 1.5 IQR from the nearer quartile), are considered outliers and are plotted individually.
