# Supplementary material for: Rituximab versus azathioprine for maintenance of remission for patients with ANCA-associated vasculitis and relapsing disease: an international randomised controlled trial
Source: Ann Rheum Dis. 2023 Mar 23;82(7):937–44. doi: 10.1136/ard-2022-223559 (PMC10313987; doi:10.1136/ard-2022-223559)
Supplement: Supplementary data [file ard-2022-223559supp004.pdf]

Supplementary Figure 4: Time to first severe adverse event in the RITAZAREM trial

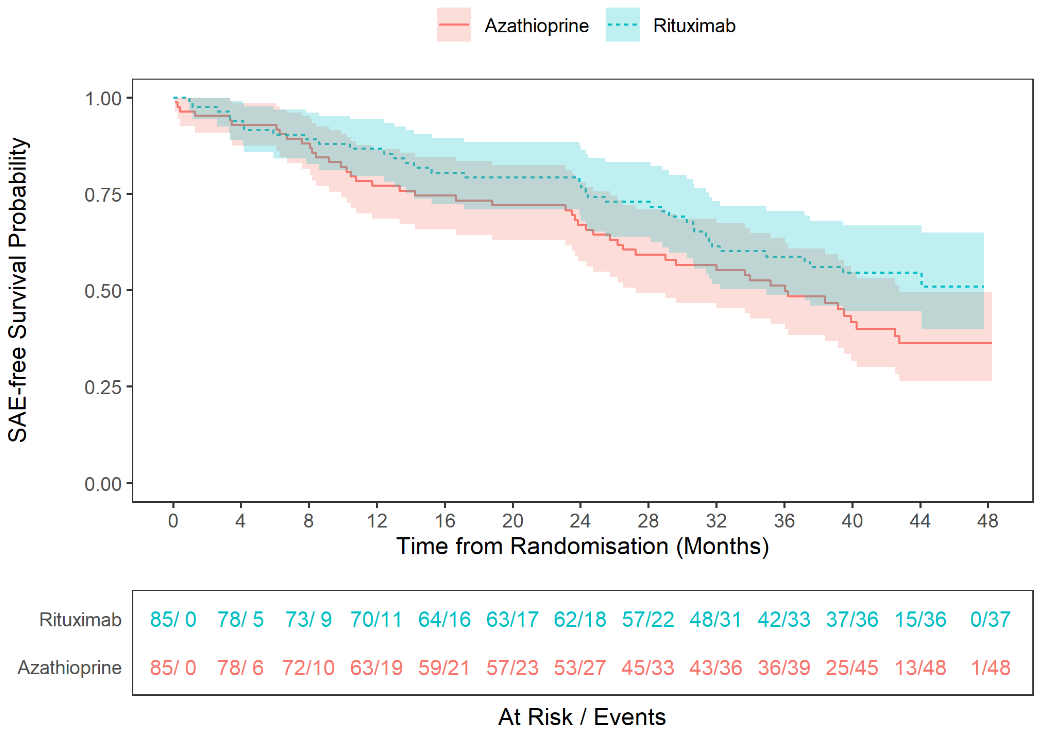

Shaded areas represent 95% confidence intervals.
