# Supplementary material for: Rituximab versus azathioprine for maintenance of remission for patients with ANCA-associated vasculitis and relapsing disease: an international randomised controlled trial
Source: Ann Rheum Dis. 2023 Mar 23;82(7):937–44. doi: 10.1136/ard-2022-223559 (PMC10313987; doi:10.1136/ard-2022-223559)
Supplement: Supplementary data [file ard-2022-223559supp002.pdf]

Supplementary Figure 2: Physical Component Scores and Mental Component Scores of SF-36 during the RITAZAREM trial per treatment group

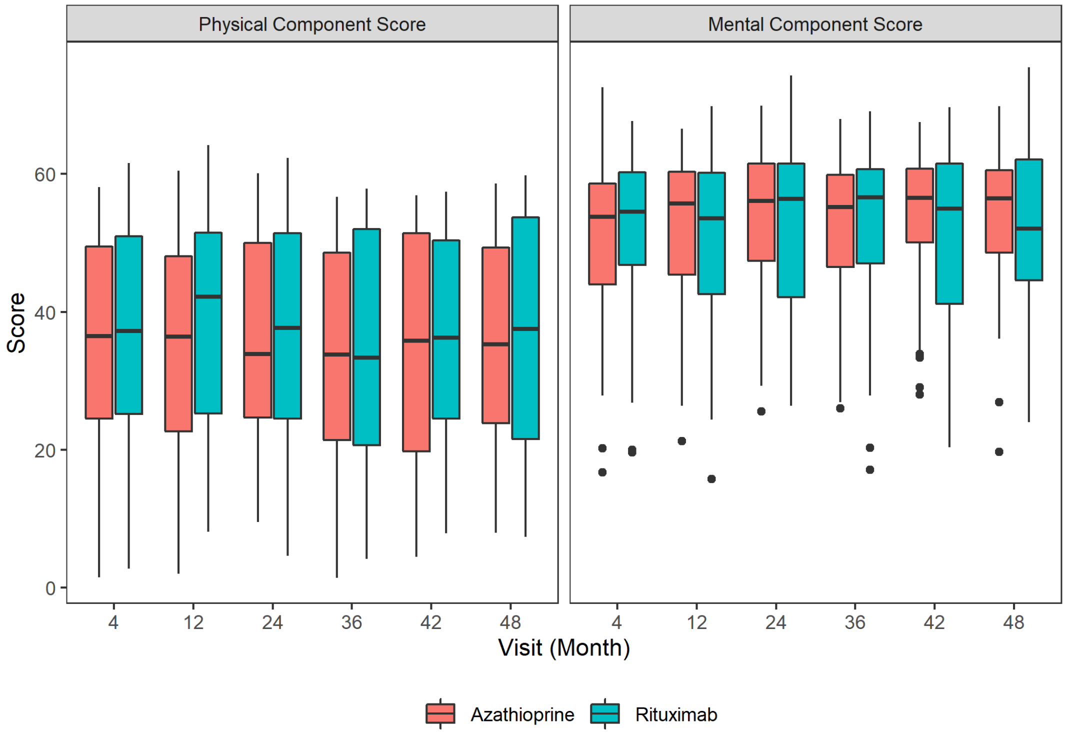

Boxes show the interquartile range (IQR) and horizontal line inside the box shows the median. The two whiskers on either side of the box represent values, up to and including, 1.5 times the length of the IQR. Values outside the range of the whiskers (more than 1.5 IQR from the nearer quartile), are considered outliers and are plotted individually.
